# A cross-sectional analysis of perinatal depressive symptoms among Punjabi-speaking women: are they at risk?

**DOI:** 10.1186/s12884-015-0568-2

**Published:** 2015-07-22

**Authors:** Raman Sanghera, Sabrina T. Wong, Helen Brown

**Affiliations:** Fraser Health Authority, Public Health Nurse, Newton Public Health Unit, #200 7337 137th Street, Surrey, BC V3W 1A4 Canada; University of British Columbia School of Nursing and Centre for Health Services and Policy Research, Co-Director of BC node of the Canadian Primary Care Sentinel Surveillance Network, T201 2211 Wesbrook Mall, Vancouver, BC V6T 2B5 Canada; University of British Columbia School of Nursing, T149-2211 Wesbrook Mall, Vancouver, BC V6T 2B5 Canada

**Keywords:** Pregnancy, South Asian, Ethnicity, Health disparity, Immigrant, Canada primary health care, public health

## Abstract

**Background:**

Depression is the leading cause of disability for childbearing women. We examined three specific research questions among Punjabi-speaking women residing in the Fraser Health Authority: 1) What are the prevalence rates of prenatal depressive symptoms? 2) Do Punjabi-speaking women have a higher likelihood of reporting depressive symptoms compared to English-speaking women after controlling for age, level of education and financial worries, and 3) Given the same level of exposure to level of education and financial worries, do Punjabi-speaking women have the same likelihood of reporting depressive symptoms?

**Methods:**

Data originated from the Fraser Health Authority prenatal registration database consisting of pregnant women (n = 9684) who completed a prenatal registration form between June 2009 and August 2010; 9.1 % indicated speaking Punjabi. The Whooley Depression Screen measured depressive symptoms. Chi-square tests and logistic multiple regression were used to examine the rates of reporting depressive symptoms among Punjabi-speaking women compared to English-speaking women.

**Results:**

Punjabi-speaking women are at a higher risk for perinatal depressive symptoms. Women needing an interpreter were more likely to report prenatal depressive symptoms compared to English-speaking women. All registrants who reported financial worries had four and a half times the odds of reporting depressive symptoms. The impact of financial worries was significantly greater in the English-speaking women compared to the Punjabi-speaking women needing an interpreter.

**Conclusion:**

Using an established screening device, Punjabi-speaking women were found to be at higher risk for prenatal depressive symptoms.

## Background

Depression is the leading cause of disability for childbearing women [1]. The term ‘perinatal’ depression encompasses prenatal and/or postpartum depression within the first year, which often arises during pregnancy and extends into the postpartum period [1]. The exact cause of perinatal depression remains unclear, though evidence suggests that depression in pregnancy is positively associated with postpartum depression [2–5]. Past work also indicates that perinatal depression results from a combination of biological and psychosocial factors. A history of mental illness or anxiety, the lack of social support, and stressful life events are the strongest predictors of depression within the perinatal period [4–7]. Poor marital relationship, socioeconomic status, obstetric complications, and in particular, migration experience have also been shown to influence the onset of depressive symptoms in women in the perinatal period [6–10].

There are not only detrimental impacts of perinatal depression to women and new mothers but also on infants [2, 6]. Depression during pregnancy is positively associated with fetus growth delays, preeclampsia, and premature delivery [1]. Neonates born to mothers experiencing depression may have low birth weights, be less responsive to stimulation and have low vagal tone [2, 6].

The reported prevalence of perinatal depression tends to vary widely because of the manner in which the depression is defined, how the depression is diagnosed, characteristics of the population being studied, and the period of time being considered [11]. Generally prevalence rates for both prenatal and postpartum depression are similar, with a commonly reported estimate of 13 % [3, 6, 7, 12, 13]. Rates of depression appear higher in immigrant women when compared to Canadian-born women [14–17]. Stewart [16] and Sword [17] found that immigrant women were more likely to report depressive symptoms. Similarly, Collins, Zimmerman, & Howard [18] and Fung & Dennis [19] determined that immigrant women had a high risk of depression with a prevalence range of 24 to 42 %.

Until recently, perinatal depression was typically viewed as a Western phenomenon. Recent research, however, indicates depression associated with childbirth has detrimental consequences for women and families from many nations and cultures [3]. In British Columbia (BC), Canada one in five women are estimated to experience significant depression related to pregnancy and childbirth; [1] Immigrant women are at a high risk for perinatal depression due to numerous stressors associated with migration and acculturation [15–17]. Limited research has been undertaken to understand the presence of perinatal depressive symptoms, specifically among Punjabi-speaking women living in Canada. Thus, it is unknown whether these women are at a higher *risk* for prenatal depression than their English-speaking counterparts.

The purpose of this study was to gain a better understanding of perinatal depressive symptoms among Punjabi-speaking women, all of whom are assumed to be South Asian. We examined three specific research questions among Punjabi-speaking women residing in the Fraser Health Authority (FHA): 1) What are the prevalence rates of prenatal depressive symptoms? 2) Do Punjabi-speaking women have a higher likelihood of reporting depressive symptoms than English-speaking women after controlling for level of education and financial worries? and 3) Given the same level of exposure to level of education and financial worries, do Punjabi-speaking women have the same likelihood of reporting depressive symptoms?

## Methods

We used a cross-sectional descriptive study design and completed secondary analyses of a database registered in the Best Beginnings program. Pregnant South Asian women, those who self-identify as being associated with any southern part of Asia or South Asian group, [20] were the group of interest given that individuals from India account for the largest proportion of all South Asian immigrants in Canada [21]. One of the most densely populated areas of FHA, British Columbia is the city of Surrey, where India is the most common country of birth and Punjabi is the most common language spoken at home next to English among immigrants [21]. Speaking Punjabi was taken to be an indicator of being South Asian in this study since this is the most common region of immigrants’ birthplace within Surrey. Surrey has the largest live birth rate in the entire Fraser Health Authority; South Asian immigrants account for a large proportion of these births [22–24].

### Participants

The Best Beginnings Program located in the FHA provides public health services for pregnant and postpartum women and babies up to two years of age [25]. Women who register for this program self-report whether they speak Punjabi or another language the most. Given the small number of women who reported speaking any other South Asian language, those who were included in our study were: Punjabi- and English-speaking who registered for the Best Beginnings Program by completing a prenatal registration form. We examined two groups of Punjabi-speaking women; one group included pregnant women who spoke Punjabi and indicated they required an interpreter and the other group were pregnant women who were Punjabi-speaking but did not indicate a need for an interpreter. We also included English-speaking pregnant women who did not indicate a need for an interpreter. Our rationale for the English-speaking group was that regardless of ethnicity, this group would contain women who were more accustomed to living in Canada.

### Best beginnings database

The prenatal database was developed using information collected in the Best Beginnings registration form which includes self-reported information on depressive symptoms, level of education, financial worries, and tobacco use. Roughly 40 % of pregnant women registered for the program throughout FHA. However, the registration rates varied widely from one health unit to another with registration rates being higher in areas where existing prenatal registration programs were in place. In the FHA where the majority of Punjabi-speaking women live (Surrey) the registration rate is closer to 70 %. This higher registration rate reflects the comprehensive nature of the prenatal registration program in this jurisdiction. On average, women registered at 27 weeks gestation and only 15 % registered prior to 20 weeks [26].

Screening for depressive symptoms was measured using the Whooley Depression Screen. Primary language and need for interpreter was also assessed. The prenatal registration form is completed through a local health unit, physician office, local hospital, or online at any time during a woman’s pregnancy. Punjabi translations of the form were available upon request. The purpose of the registration form was to identify pregnant women who could be at risk for depression, tobacco exposure, and other potential vulnerabilities (e.g. financial need) [26]. All completed prenatal registration forms were sent to health units for determination of follow-up by public health nurses (PHNs).

We conducted a secondary analysis of the 2009-2010 FHA prenatal registration database. The data from the prenatal registration forms was entered into a regional database developed by Fraser Health specialists [26]. During this time period, approximately 9684 women completed the prenatal registration form. We compared the prenatal database to Fraser Health Profiles to examine whether the pregnant women in the database was similar to the women in Fraser Health. Women who primarily spoke Punjabi made up 9 % of the prenatal registration data compared to 7 % of the population living in Fraser Health [22]. Among the prenatal registrants, 15.2 % indicated worrying about finances, while the Fraser Health census data [22] reported that 14.3 % of its residents are low income. Eleven percent of prenatal registrants reported some high school, which is lower than the Fraser Health census data, [22] where 17 % reported they have completed less than high school.

### Variables

The independent variable of interest was Punjabi- versus English-speaking. The confounding variables included: age, level of education (some high school, high school, some college or university, college or university), and financial worries. The dependent variable of interest was presence of depressive symptoms as measured by the Whooley Depression Screen. This screen is a reliable and valid measure consisting of two questions: “During the past month have you often been bothered by feeling down, depressed or hopeless?” and “During the past month have you often been bothered by little interest or pleasure in doing things?” Research indicates these two questions address mood and interests, are as likely to be effective in detecting depressive symptoms compared to other depressive symptom screening tools, and are feasible for routine use in routine health care [27, 28]. The tool is only used for screening purposes and not a diagnostic instrument for depression [27, 28].

The Whooley Depression Screen is a two-item screening tool considered to be simple, quick, and efficient for screening for depressive symptoms during pregnancy [28]. Whooley *et al.*, [28] compared the validity of the Whooley Depression Screen to six previously validated instruments including two types of the Centre for Epidemiological Studies Depression Scale (CES-D), two forms of the Beck Depression Inventory (BDI), the Medical Outcome Study (MOS), and the Symptom-Driven Diagnostic System for Primary Care (SDSS-PC). These two questions are considered separate items; a negative response to both questions on the screening tool indicates that depression is highly unlikely [28], A positive response to either one of these items in the Whooley Depression Screen indicates the presence of depressive symptoms. The calculated sensitivity of 96 % and specificity of 57 % was based on self-report [28].

### Data analysis

We conducted a power analysis using the statistical program R, version 2.15, to determine sample size adequacy for detecting a statistically significant difference in prenatal depressive symptoms between Punjabi- and English-speaking women. Based on an effect size of 0.35, [15, 29] our sample size for the Punjabi-speaking (n = 887) and English-speaking (n = 7423) groups provided a power of 1 in a two-tailed test at a significance level of 0.05. Punjabi-speaking women were divided into two groups: those who indicated they needed an interpreter and those who did not. A combination of parametric (e.g., analysis of variance) and non-parametric (e.g., Chi-square) tests was used to examine if there were differences between the groups. We conducted a series of logistic regressions to examine whether the statistically significant association between language and presence of depressive symptoms was attenuated by age, level of education, and financial worries. The Statistical Package for Social Sciences (SPSS), version 20, was used for the analyses. The University of British Columbia Behavioural Ethics Research Ethics Board and Fraser Health Research Ethics Board approved of all procedures.

## Results

The characteristics of the Punjabi- and English-speaking prenatal registrants are summarized in Table 1. English-speaking registrants were significantly older than the Punjabi-speaking registrants and the Punjabi-speaking registrants with an interpreter (p < 0.05). A significant difference was found in the reported level of education between the three groups (*X*^2^ = 563.32, p < 0.001). Many more Punjabi-speaking women needing an interpreter had not completed high school compared to those who were English-speaking (43 % versus 10 %, respectively). Almost three-quarters (74.5 %) of the English-speaking women reported to have partially or fully completed some college or university, compared to 36 % of Punjabi-speaking women needing an interpreter. A similar percentage of women in both the Punjabi-speaking women needing an interpreter and the English-speaking group reported having financial worries.

**Table 1:** Characteristics of prenatal registrants

	Punjabi-speaking with interpreter n = 556	Punjabi-speaking n = 331	English-speaking n = 7423
Age: mean (sd)*	29.0 (4.5)	29.1 (4.2)	29.6 (5.2)
Education: n (%)*			
Some high school	196 (42.6)	80 (28.8)	667 (9.6)
High school graduation	100 (27.7)	45 (16.2)	1112 (15.9)
Some college/university	67 (14.6)	51 (18.3)	1688 (24.2)
College/university graduation	97 (21.1)	102 (36.7)	3506 (50.3)
Financial worries:n (%)*	81 (15.3)	28 (8.9)	1151 (15.6)

Note. *p < .0.05

Table 2 shows that the two Punjabi-speaking groups reported a higher prevalence of depressive symptoms compared to the English-speaking group (*X*^2^ = 47.50, p = 0.001). Significantly more women needing an interpreter reported depressive symptoms than the Punjabi-speaking and English-speaking women. Even after controlling for age, level of education, and financial worries, the women needing an interpreter had twice the likelihood of reporting depressive symptoms compared with their English-speaking counterparts (Table 3). Prenatal registrants who reported financial worries were 4.5 times more likely to report depressive symptoms. Registrants with an incomplete high school education were two times more likely to report depressive symptoms than those registrants who had completed their post-secondary education.

**Table 2:** Depressive symptoms in fraser health prenatal registrants

	Punjabi-speaking with interpreter n = 556 n (%)	Punjabi-speaking n = 331 n (%)	English-speaking n = 7423 n (%)
Yes*	109 (20.6)	42 (13.1)	799 (10.8)

Note. *p < 0.05

**Table 3:** Factors associated with depressive symptoms in Fraser Health Prenatal Registrants

Factor	Odds ratio (95 % CIs)
Ethnic/Language group	
Punjabi-speaking with interpreter*	1.99 [1.54-2.59]
Punjabi-speaking	1.29 [0.88-1.90]
English-speaking (ref)	1
Age	0.97 [0.98-1.01]
Financial worries	
Yes*	4.57 [3.90-5.35]
No (ref)	1
Education	
Some high school*	2.03 [1.62-2.05]
High school graduation*	1.29 [1.04-1.60]
Some college/university*	1.35 [1.11-1.63]
College/university graduation (ref)	1

Note. *p < 0.05

We conducted a logistic regression of Punjabi-speaking women who needed an interpreter (Table 4) in order to examine whether the presence of age, level of education, and financial worries was associated with the reporting of depressive symptoms. Our results suggest that those reporting financial worries were two times more likely of also reporting depressive symptoms. Educational level had no statistically significant association in the reporting of depressive symptoms in the interpreter group; however, the point-estimates indicate that education on all three levels is still associated with the reporting of depressive symptoms within this group. The point estimates are suggestive of associations that are almost similar, if not stronger, in magnitude, than the English-speaking women group (Table 5). Those who have only completed high school graduation appeared to have almost 2.5 times the odds of reporting depressive symptoms than those who had graduated from college.

**Table 4:** Factors associated with depressive symptoms in Punjabi-speaking with Interpreter Women

Factor	OR (95 % CIs)
Age	1.03 [0.98-1.09]
Financial worries*	2.06 [1.17-3.61]
Education	
Some high school	1.49 [0.76-2.92]
High school graduation	1.96 [0.94-4.08]
Some college/university	1.67 [0.74-3.79]
College/university graduation (ref)	1

Note. *p < 0.05

**Table 5:** Factors associated with depressive symptoms in English-Speaking Women

Factor	OR (95 % CIs)
Age	1.00 [0.98-1.01]
Financial worries*	5.03 [4.25-5.94]
Education	
Some high school*	2.47 [1.92-3.17]
High school graduation	1.24 [0.94-4.08]
Some college/university*	1.37 [1.12-1.67]
College/university graduation (ref)	1

Note. *p < 0.05

Financial worries was the strongest associated factor in the reporting of depressive symptoms among the English-speaking women. The impact of financial worries was significantly greater in the English-speaking women compared to the Punjabi-speaking women with interpreter group. English-speaking women reporting financial worries had 5 times the likelihood of reporting depressive symptoms. In addition, within this group, education was significantly associated with the reporting of depressive symptoms. Notably, English-speaking women who had completed only partial high school education had more than twice the odds of reporting depressive symptoms than those who had completed post-secondary education.

## Discussion

This study is one of the few Canadian studies to investigate perinatal depressive symptoms among Punjabi-speaking women. The findings suggest that pregnant South Asian women who are Punjabi-speaking are at a higher risk for experiencing perinatal depressive symptoms. Unique to this study are the findings that being a Punjabi-speaking woman with poor English language skills is strongly associated with self-reporting of depressive symptoms compared to being an English-speaking woman. Our findings are similar to studies completed in countries that suggest immigrant women are at a higher risk of prenatal depression than non-immigrant women [21–23]. It is likely that the women needing an interpreter are more recent immigrants to Canada who still hold strong cultural beliefs and values associated with their country of origin, India.

Depression in pregnancy is highly prevalent in immigrant women without social support [30, 31]. The process of migration and the associated impacts of language, gender and cultural environments intersect to create circumstances that increase the risks for perinatal depression for these women [10, 31]. Migrating to Canada for South Asian women can lead to a separation of social supports and cultural norms such as the lack of support from their mothers and female relatives. Moreover, the increased dependency on their spouses and inability to financially contribute to the family could also be additional risk factors for developing depressive symptoms [31].

### Financial uncertainties in context

Worrying about finances was significantly associated with reporting depressive symptoms in pregnancy. Our results are somewhat surprising since previous studies on prenatal depression have found immigrant women are more likely to have a low income compared to Canadian-born women [14, 15]. More work needs to be completed since financial uncertainties affect both English- and Punjabi-speaking women’s reports of depressive symptoms. What is known is that financial uncertainties can negatively affect the mental health of immigrants [15, 32]; in FHA many of these immigrants could be Punjabi-speaking women, whom often experience employment challenges and economic insecurities throughout the immigration period [33].

Employment is often a challenging adjustment for Punjabi-speaking women, since many may not have worked in their country of origin, relying on men to be the primary income earners [32]. In Canada these women may have to work outside the home and are often still expected to fulfill culture-specific gender roles within the home. Patriarchal practices within the family can result in greater stress and responsibility for Punjabi-speaking women, [34–36] increasing their risk for depression during the sensitive perinatal period. Since their income is necessary for the household, many women are reluctant to take leave for self-care [32]. Male partners may also be forced to work strenuously and may be unavailable for support to their wives during the prenatal and postpartum periods. Some women also face pressure to save their earnings to sponsor their spouse or other family members who remain in their country of origin. These circumstances illustrate how the cultural context of women’s lives can be shaped by the intersection of patriarchal practices, marital relationships, and economic realities of new immigrant women and their families. These dynamics affect the lives of women in specific ways during pregnancy. These findings indicate that depressive symptoms in pregnancy could provide an entry point for beginning to address the various factors in promoting health pregnancy outcomes.

Reporting being worried about finances also had a strong association with the reporting of prenatal depressive symptoms among English-speaking women, especially compared to the Punjabi-speaking women needing an interpreter. Past work suggests that socio-economic status, regardless of language spoken, is associated with depressive symptoms in perinatal depression [6, 12].

### Education in context

In the overall regression model of the entire sample of prenatal registrants, the association of education with the reporting of depressive symptoms was statistically significant. In the separate regression analysis; however, education was not statistically associated with prenatal depressive symptoms in the Punjabi-speaking with interpreter group, which may be attributed to a smaller sample size and a lack of heterogeneity in the education levels within this group. Nonetheless the results suggest that women with less education are more likely to report depressive symptoms in pregnancy. Certainly, the opportunities for obtaining employment are lower for those who have less education and cannot speak English; finances, as a consequence, are more likely to be a concern for these women. The affiliation to their home country’s culture may be stronger for those who indicate a need for an interpreter, likely because they are more recent immigrants to Canada [37]. Thus, cultural affiliation should be analyzed in conjunction with migration [38]. Language spoken inside and outside the home can also be linked to the progressive integration into the community at large [37].

#### Limitations

The results of this study should be interpreted with caution. The data are cross-sectional and do not offer insight into the nature of perinatal depression over time or the timing of depressive symptoms among these women. Although the data captures one of the largest samples of Punjabi-speaking pregnant women in Canada, the findings have limited generalizability since only data from FHA is included and the precise proportion of pregnant women living in Fraser Health at the time who were registered in the Best Beginnings program is unknown. The secondary data are from 2009 to 2010; after this period, no central database was available. The prenatal registration form is a self-report form and the screening is completed on self-report measures. Some Punjabi-speaking women may have misunderstood the Whooley Depression Screen questions since depressive symptoms within the South Asian culture is often somatised, and physical distress may be combined with mental distress [38]. The two questions of the tool have not been validated in Punjabi-speaking women. Moreover, the specificity of the tool suggests there is a 57 % potential to misclassify women’s depressive symptoms. The prenatal registration forms also did not assess for the presence of pre-existing mental illnesses; hence, it is unknown whether these depressive symptoms arose in the perinatal period or were due in part to recurrent and untreated depression or other mental illnesses. Finally, in the case of the English as a primary language group, it is likely that this group included other ethnic/cultural backgrounds and second generation immigrants that have their own vulnerabilities to depression.

Despite these limitations, the study findings suggest that through screening, targeting, and accounting for the social determinants, nurses and other healthcare providers could work towards the overall prevention and early treatment of depressive symptoms with Punjabi-speaking pregnant women. The findings suggest that a prenatal registration database that contains intersecting dimensions of language, culture, migration, education, and financial concerns maintained by a health authority could assist in identifying women who may be at risk for developing ill health. Obtaining data on language and migration in addition to other social determinants of health is important for developing gender and culturally sensitive public health interventions to reduce the health disparities in different groups. In particular, close attention must be given to the reporting of financial worries, independent of language spoken. Based on identified risk factors, public health nurses and other health professionals could work on developing stronger collaborations to meet the mental healthcare needs of Punjabi-speaking women.

These findings provide some evidence of the need to allocate more targeted resources and funds for preventive and treatment options for populations who speak English as a second language. More work is needed to examine what tools and programs are needed to identify and support Punjabi-speaking women’s mental health care needs.

## Conclusion

Using an established screening device, Punjabi-speaking women were found to be at higher risk for prenatal depressive symptoms. Further studies are needed to explore the prevalence of perinatal depression among Punjabi-speaking and additional groups of South Asian immigrant women with additional validated instruments. New research should continue to evaluate the effectiveness of depression screening measures for women in the perinatal period to determine which measures are more culturally sensitive for a given population group and setting.

This study provides insight into the context of depressive symptoms in pregnancy for Punjabi-speaking women. Additional longitudinal studies would be needed to examine maternal and fetal outcomes and to consider the process of perinatal depression during pregnancy and into the postpartum period. Further examination of the intersecting risk factors and their relation to the recovery process of perinatal depression may reveal valuable findings about the reproductive mental healthcare needs of women.

## References

[1] BC reproductive mental health program: BC Women’s Hospital and Health Centre. Addressing Perinatal Depression: A Framework for BC’s Health Authorities. Vancouver, Canada; 2006:1–40.

[2] Field T (2011). Prenatal depression effects on early development: a review. Infant Behav Dev.

[3] Milgrom J, Gemmill AW, Bilszta JL, Hayes B, Barnett B, Brooks J, Ericksen J, Ellwood D, Buist A (2008). Antenatal risk factors for postnatal depression: a large prospective study. J Affect Disord.

[4] Reid H, Power M, Cheshire K (2009). Factors influencing antenatal depression, anxiety and stress. Br J Midwifery.

[5] Oppo A, Mauri M, Ramacciotti D, Camilleri V, Banti S, Borri C, Rambelli C, Montagnani MS, Cortopassi S, Bettini A, Ricciardulli S, Montaresi S, Rucci P, Beck CT, Cassano GB (2009). Risk factors for postpartum depression: the role of the Postpartum Depression Predictors Inventory-Revised (PDPI-R). Results from the Perinatal Depression-Research & Screening Unit (PNDReScU) study. Arch Womens Ment Health.

[6] Leigh B, Milgrom J (2008). Risk factors for antenatal depression, postnatal depression and parenting stress. BMC Psychiatry.

[7] O’hara MW, Swain AM (1996). Rates and risk of postpartum depression—a meta-analysis. Int Rev Psychiatry.

[8] Beck CT (2006). Postpartum depression: it isn’t just the blues. Am J Nurs.

[9] Green J (1998). Postnatal depression or perinatal dysphoria? Findings from a longitudinal community-based study using the Edinburgh Postnatal Depression Scale. J Reprod Infant Psychol.

[10] O’Mahony J, Donnelly T (2010). Immigrant and refugee women’s post-partum depression help-seeking experiences and access to care: a review and analysis of the literature. J Psychiatr Ment Health Nurs.

[11] Gjerdingen D, Crow S, McGovern P, Miner M, Center B (2011). Changes in depressive symptoms over 0-9 months postpartum. J Womens Health (Larchmt).

[12] Beck CT (2002). Postpartum depression: a metasynthesis. Qual Health Res.

[13] Sobey WS (2002). Barriers to postpartum depression prevention and treatment: a policy analysis. J Midwifery Womens Health.

[14] Mechakra-Tahiri S, Zunzunegui MV, Seguin L (2007). Self-rated health and postnatal depressive symptoms among immigrant mothers in Québec. Women Health.

[15] Miszkurka M, Goulet L, Zunzunegui MV (2010). Contributions of immigration to depressive symptoms among pregnant women in Canada. Can J Public Heal.

[16] Stewart DE, Gagnon A, Saucier J-F, Wahoush O, Dougherty G (2008). Postpartum depression symptoms in newcomers. Can J Psychiatry.

[17] Sword W, Watt S, Krueger P (2006). Postpartum health, service needs, and access to care experiences of immigrant and Canadian-born women. J Obstet Gynecol Neonatal Nurs.

[18] Collins CH, Zimmerman C, Howard LM (2011). Refugee, asylum seeker, immigrant women and postnatal depression: rates and risk factors. Arch Womens Ment Health.

[19] Fung K, Dennis C-L (2010). Postpartum depression among immigrant women. Curr Opin Psychiatry.

[20] Tran K, Kaddatz J, Allard P. South Asians in Canada: unity through diversity. Can Soc Trends. 2005:20–25.

[21] Statistics Canada. 2006 Census: immigration and citizenship. Ottawa, ON; 2007.

[22] Health and Business Analytics Fraser Health Authority. Health profile 2011: a snapshot of the health of fraser health residents. Surrey, BC; 2011.

[23] City of Surrey (2009). Citizenship and Immigration Fact Sheet.

[24] The South Asian Community in Canada [http://www.statcan.gc.ca/pub/89-621-x/89-621-x2007006-eng.htm]

[25] Fraser Health: Best Beginnings [http://bestbeginnings.fraserhealth.ca/default.aspx]

[26] Prenatal Registration: Support for a healthy pregnancy and baby [http://www.fraserhealth.ca/your_health/best-beginnings/pregnancy/prenatal-registration/register-your-pregnancy-with-us]

[27] Arroll B, Khin N, Kerse N (2003). Screening for depression in primary care with two verbally asked questions: cross sectional study. BMJ.

[28] Whooley MA, Avins AL, Miranda J, Browner WS (1997). Case-finding instruments for depression. Two questions are as good as many. J Gen Intern Med.

[29] Zelkowitz P, Schinazi J, Katofsky L, Saucier JF, Valenzuela M, Westreich R, Dayan J (2004). Factors associated with depression in pregnant immigrant women. Transcult Psychiatry.

[30] Miszkurka M, Goulet L, Zunzunegui MV (2012). Antenatal depressive symptoms among Canadian-born and immigrant women in Quebec: differential exposure and vulnerability to contextual risk factors. Soc Psychiatry Psychiatr Epidemiol.

[31] Tewary S (2005). Asian Indian immigrant women: a theoretical perspective on mental health. J Hum Behav Soc Environ.

[32] Ahmad F, Shik A, Vanza R, Cheung AM, George U, Stewart DE (2005). Voices of South Asian Women: immigration and mental health. Women Health.

[33] Mehrotra M, Calasanti TM (2010). The family as a site for gendered ethnic identity work among Asian Indian immigrants. J Fam Issues.

[34] Choudhry UK (2001). Uprooting and resettlement experiences of South Asian immigrant women. West J Nurs Res.

[35] Ralston H (1991). Race, class, gender and work experience of South Asian immigrant women in Atlantic Canada. Can Ethn Stud.

[36] Abraham M (2005). Domestic violence and the Indian diaspora in the United States. Indian J Gend Stud.

[37] Vissandjee B, Desmeules M, Cao Z, Abdool S, Kazanjian A (2004). Integrating ethnicity and migration as determinants of Canadian women’s health. BMC Womens Health.

[38] Fenton S, Sadiq-Sangster A (1996). Culture, relativism and the expression of mental distress: South Asian women in Britain. Sociol Heal Illn.

